# The role of Panax notoginseng saponins in cerebrovascular neurological disorders: an overview of mechanisms and functions

**DOI:** 10.3389/fphar.2025.1693253

**Published:** 2025-11-13

**Authors:** Bin Wang, Xu Gao, Yali Zhang, Yuhua Xiao, Tong Chen, Zhouying Shi, Yue Yuan, Ping Li

**Affiliations:** 1 College of Traditional Chinese Medicine, Changchun University of Chinese Medicine, Changchun, China; 2 College of Basic Medicine, Changchun University of Chinese Medicine, Changchun, China; 3 College of Nursing, Changchun University of Chinese Medicine, Changchun, China

**Keywords:** Panax notoginseng saponins, cerebrovascular, neurological diseases, pharmacologiceffects, mechanism of action

## Abstract

Cerebrovascular neurological disorders, especially high-mortality and disabling stroke subtypes such as ischemic stroke and hemorrhagic stroke, have become a major global health issue. In addition to conventional treatments, the role of herbal medicines and their active ingredients in the prevention and treatment of cerebrovascular and nervous system diseases has received increasing attention in recent years. Among them, the primary active ingredient of Panax notoginseng is Panax Notoginseng Saponins (PNS), has become a research hotspot due to its diverse pharmacological activities. Existing evidence suggests that PNS exhibits various effects including anti-inflammatory, antioxidant, anti-apoptotic, immune regulation, neuroprotection, blood sugar and lipid lowering, and cardiovascular protection. This review systematically searches multiple databases for literature related to PNS and cerebrovascular neurological disorders, focusing on summarizing the role of PNS in specific diseases such as ischemic stroke, hemorrhagic stroke, and neurodegenerative disorders, exploring its pharmacokinetic characteristics, main mechanisms of action, and clinical application prospects, aiming to provide a theoretical basis for the in-depth research and development of PNS in cerebrovascular neurological disorders.

## 1 Introduction

The incidence of neurological diseases is increasing year by year. These diseases are caused by many endogenous (genetic, metabolic, immune, etc.) and exogenous factors (trauma, environment, lifestyle, etc.) (Mehndiratta and Aggarwal, 2021), mainly neurodegenerative and cerebrovascular diseases, which are the second leading cause of death worldwide in addition to cardiovascular diseases, including stroke Alzheimer’s disease (AD), Parkinson’s disease, and depression are the most common neurological disorders, and their pathogenesis is complex, there is no effective cure or prevention strategy, and the efficacy and side effects of related therapeutic drugs change with the prolongation of the disease. As an effective multi-targeted therapy, traditional Chinese medicine, as an effective multi-targeted therapy, plays an important role in treating symptoms and slowing disease progression.

Panax notoginseng is the dried root and rhizome of the plant of the Araliaceae, which has the effects of stopping bleeding and dispersing blood stasis, subduing swellings and relieving pain, and is one of the most widely used Chinese herbs in China and other countries mainly for the treatment of cardiovascular and cerebral vascular diseases, such as coronary artery disease, atherosclerosis and cerebral infarction (Li et al., 2020). At present, more than 200 compounds have been isolated from Panax notoginseng, mainly including saponins, flavonoids and polysaccharides, etc. PNS is a class of chemical mixtures containing different dammarane-type saponins extracted from Panax notoginseng, including PNS (R1-R6) and PNS (VII); different parts of Panax notoginseng (roots, stems, leaves, and flowers) contain different saponins, such as Ginsenoside (Rb1, Re, Rg1, Rg2, Rh1) (Zhou et al., 2017; Sun et al., 2023), and currently there are more than 180 PNS isolated and identified from various parts of Panax notoginseng, based on the structure of the saponins, the saponins can be classified into five main types, the most important of which are the protopanaxadiol type (PPD) and protopanaxatriol type (PPT) (Yang et al., 2014; Wang et al., 2016), and the content of saponins contained in different parts of the plant varies (Wei et al., 2020), and the type and amount of saponins change with the age, growth environment, and tissue type of Panax notoginseng. In addition to neurological aspects, PNS can play an important role in inhibiting inflammation, oxidative stress, apoptosis, and immunomodulation (Ling et al., 2024). It also plays an important role in the treatment of cardiovascular, diabetes, liver disease, gastrointestinal tract, bone metabolism regulation, anticancer and renal disease (Liu et al., 2020), and possesses a wide range of activities such as haemostasis, pro-angiogenesis, immunomodulation, anti-inflammation, anti-tumor, antioxidant, etc (Li W. et al., 2025). For example, PNS can further exert anti-obesity effects by reducing lipid synthesis, inhibiting adipogenesis, promoting browning of white adipose tissue, increasing energy expenditure and improving insulin sensitivity (Zhang et al., 2020).

**FIGURE 1:**
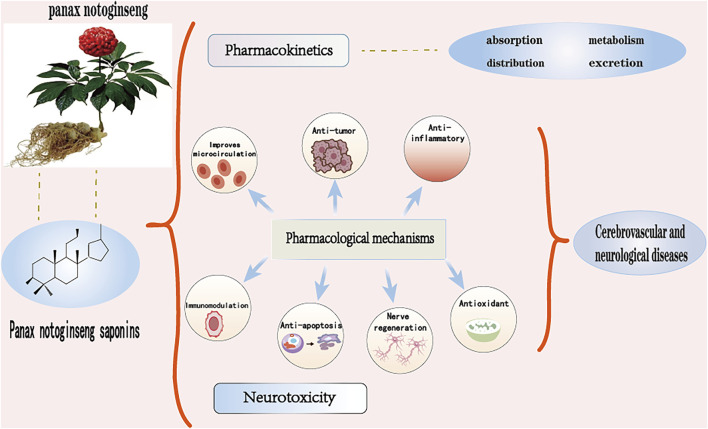
Mechanisms of PNS in the treatment of cerebrovascular neurological disorders. Partly reprinted with permission from the official website maisanqi.com.

As a novel potential therapeutic agent, herbal monomers play a unique role in the treatment of various aspects such as cancer, cardiovascular and cerebrovascular diseases (Li Z. et al., 2025). Nevertheless, besides exerting different pharmacological activities in the treatment of cerebrovascular and neurological diseases, PNS can still have some problems in the treatment of other systemic diseases and their complications, such as toxic reactions to some osteoporosis, cancer, diabetes and some drugs (Xu et al., 2019). Therefore, this paper provides a comprehensive summary of the mechanism application of PNS in cerebrovascular neurological diseases, reviewing the chemical structure, pharmacokinetics, pharmacological activity and mechanism of action in different diseases, neurotoxicity, etc., and analyzing its mechanism of action and therapeutic potential in the process of treatment, so as to improve the reference for further promotion of PNS research and application in experimental clinics (see Figure 1).

**FIGURE 2:**
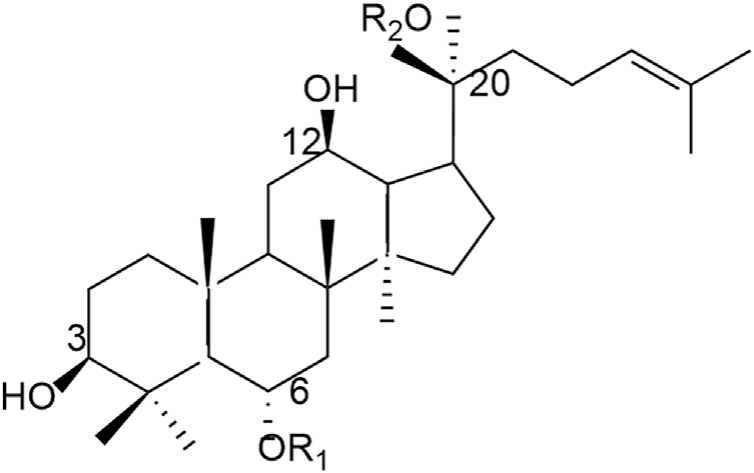
Chemical structure diagram of PNS.

As one of the main chemical components of Panax notoginseng, PNS is a dammarane-type tetracyclic triterpenoid saponin, which is mainly classified into dammarane-type 20 (S)- PPD and 20 (S)- PPT according to the hydroxyl groups attached to the glycosides (see Figure 2).

## 2 Search strategy and selection criteria

**FIGURE 3:**
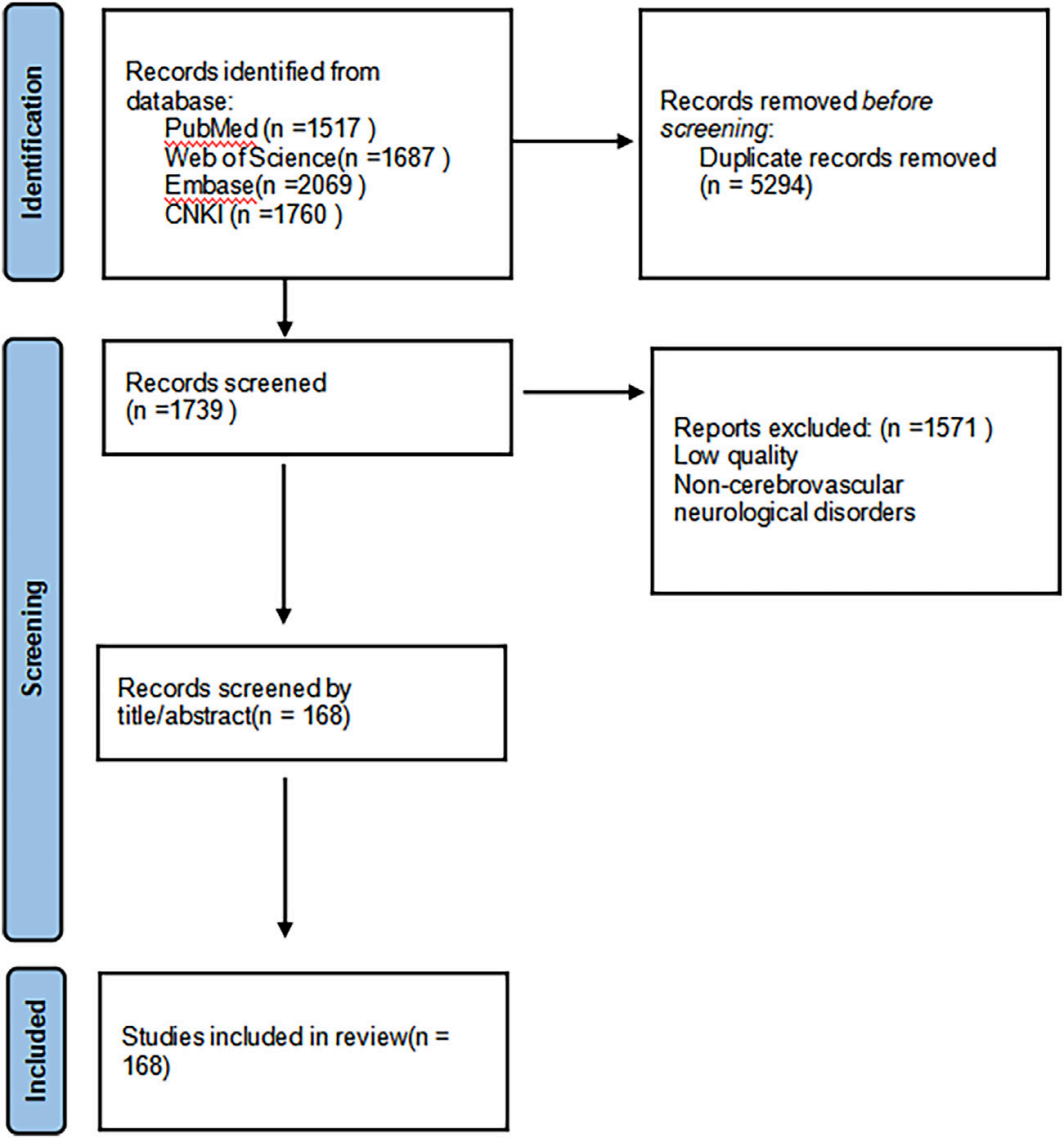
Retrieval process.

In order to comprehensively review the pharmacological effects of PNS and its therapeutic potential for cerebrovascular neurological disorders, we systematically searched multiple Chinese and English databases (including Web of Science, PubMed, Embase, and CNKI), with a time frame from the inception of each database to 31 May 2025, and supplemented the references of relevant literature through manual searches. The search strategy employed a wide range of keywords, including general terms such as “panax notoginseng saponins,” “panax notoginseng,” “mechanism of action,” “pharmacological effects,” as well as specific disease names such as “ischemic stroke,” “hemorrhagic stroke,” “Alzheimer’s disease”, “Parkinson’s disease,” and “pharmacokinetics” to ensure coverage of all aspects of its molecular mechanisms and therapeutic applications. This study established predefined inclusion criteria, primarily incorporating peer-reviewed research articles, reviews, and clinical trial reports published in English, to provide a comprehensive overview of its pharmacological characteristics; literature that did not directly relate to its pharmacological effects or was of poor quality was excluded. Detailed information about the included studies is provided in Figure 3 (see Figure 3).

## 3 Pharmacokinetics of PNS

The pharmacokinetic process of PNS *in vivo* varies from component to component, and its absorption, distribution, metabolism and excretion processes are also affected by many factors. Some natural major saponins will be released by sugar groups or dehydrated to become secondary saponins during the processing and heating of Panax notoginseng. Not only the active ingredients will be changed, but also the transformation of the compound bases makes the pharmacological effects and pharmacokinetics change (Xiong et al., 2017; Peng et al., 2018). PNS has good water solubility, low membrane permeability and high molecular weight are the main factors affecting poor bioavailability. About 90% of drug absorption mainly occurs in the small intestine, the intestinal epithelium as the main site of drug absorption, its surface mucus layer and epithelial cell membrane fluidity will have an impact on drug permeability, and the monomeric saponins in PNS are mostly large molecule water-soluble components, and when ingested orally or by gavage, these components mainly rely on the cellular bypass passive transport mechanism to be absorbed, due to the intestinal epithelial cell membrane has a due to the lipophilic nature of the intestinal epithelial cell membrane, this can prevent hydrophilic drugs from being absorbed through the transcellular pathway. In addition, PNS is easily hydrolyzed or metabolized by gut bacteria, which further affects its bioavailability. After entering the bloodstream, its distribution shows tissue specificity, but overall it is characterized by a high plasma protein binding rate and a limited volume of tissue distribution. The main metabolic pathway is mediated by hepatic cytochrome P450 enzyme system (especially CYP3A and CYP1A2) which involves hydrolysis and deglycosylation, producing active or inactive metabolites. Ultimately, the parent drug and its metabolites are excreted through the kidneys in urine, slowly eliminated from serum and tissues, with some being excreted via bile in feces, resulting in a relatively short elimination half-life (Masaoka et al., 2006; Hao et al., 2010; Kim, 2018; Li et al., 2019; Miao et al., 2022; Fu et al., 2023).

Different formulation types and delivery modes are different for the pharmacokinetic properties of PNS *in vivo* (Pang et al., 2017). The solubility and absorption of different types of saponins can be improved by adding some absorption enhancers and improving the different dosage forms of the drug, such as nanoformulations, microemulsions, enteric dissolution, etc., or by applying nano-delivery systems to increase the permeability and to combat the gastrointestinal degradation, which can largely improve the bioavailability of PNS (Ruan et al., 2010; Li Y. et al., 2018; Balusamy et al., 2023; Wang et al., 2023). Some studies have shown that the use of bioadhesive materials can improve the bioavailability of PNS in compound danshen formulations to some extent compared with other dosage forms (Chen et al., 2018); the relative bioavailability of PNS bioadhesive tablets prepared using chitosan with the main components R1, Rg1, and Rb1 was increased to 204.53% compared with ordinary tablets, respectively, 152.73%, and 150.50% (Feng et al., 2011). The lyophilization process can effectively improve the stability of PNS-loaded transfer bodies (PNS-TFSs) without affecting their transdermal absorption properties and promote faster drug absorption (Lu et al., 2024). Xuesaitong, as one of the main preparations of Panax notoginseng freeze-dried extract, has been found that the saponins in the freeze-dried Xuesaitong preparation have a high potential for drug interactions mediated by organic anion-transporting polypeptide (OATP) 1B, which can enhance its bioavailability (Pintusophon et al., 2019). The use of enteric formulations, which can release most or all of the drug at a site in the drug intestine, increases drug stability and delays the time of intestinal absorption of the drug.

Interaction with other drugs may affect their metabolism and efficacy. Ice tablet acts as an absorption enhancer, and ice tablet significantly increases the permeability of the active ingredients of PNS classes at certain molar ratios, and the degree of oral absorption of prescriptions containing ice tablet (molar ratio 1:27) was approximately three times that of the control compared to prescriptions containing no ice tablet (Kim et al., 2021). Borneol (BO) may enhance absorption and affect the distribution and metabolism of other ingredients in combination with PNS 1:1 (PNS 75 mg/kg; BO 75 mg/kg) ratio (Mei et al., 2024). A study showed that when aspirin and PNS were used together, the apparent permeability coefficient value increased significantly, and the combination of aspirin and salicylic acid was able to interfere with the function of tight junction proteins, which led to the enlargement of cellular gaps, and ultimately facilitated the uptake of drugs by PNS (Tian et al., 2018). Nanoemulsions: water-in-oil (W/O) or oil-in-water (O/W) droplets stabilized by amphiphilic surfactants, for hydrophilic macro-ingredients such as PNS, which are poorly absorbed, W/O-type nanoemulsions surfactants improve membrane fluidity and enhance the degree of transmembrane uptake, which in turn improves bioavailability (Singh et al., 2017).

In addition to oral, gavage, intravenous, and nano-delivery methods of drug administration, there are also various non-gastrointestinal mucosal delivery methods, such as nasal administration, pulmonary inhalation, and transdermal administration; these methods are all beneficial for the absorption of PNS. Nasal administration is a direct route for delivering drugs to the brain, avoiding the Blood-brain barrier (BBB), gastrointestinal degradation, and the first-pass effect in the liver (Guo et al., 2014). Through investigating the tissue distribution of drug administration in the rat’s nasal cavity and intravenous administration, the study found that after nasal administration of PNS solution, the concentration of Rg1 in the brain was significantly higher than that after intravenous injection. A major challenge currently faced in PNS research is the lack of dosage standardization; the sources of PNS extracts, the ratio of saponins, purity, and formulation types vary across different studies, making it very difficult to directly compare their pharmacokinetic parameters and efficacy. Future research urgently needs to standardize and quantify the core active components of PNS to enhance comparability across different studies.

**FIGURE 4:**
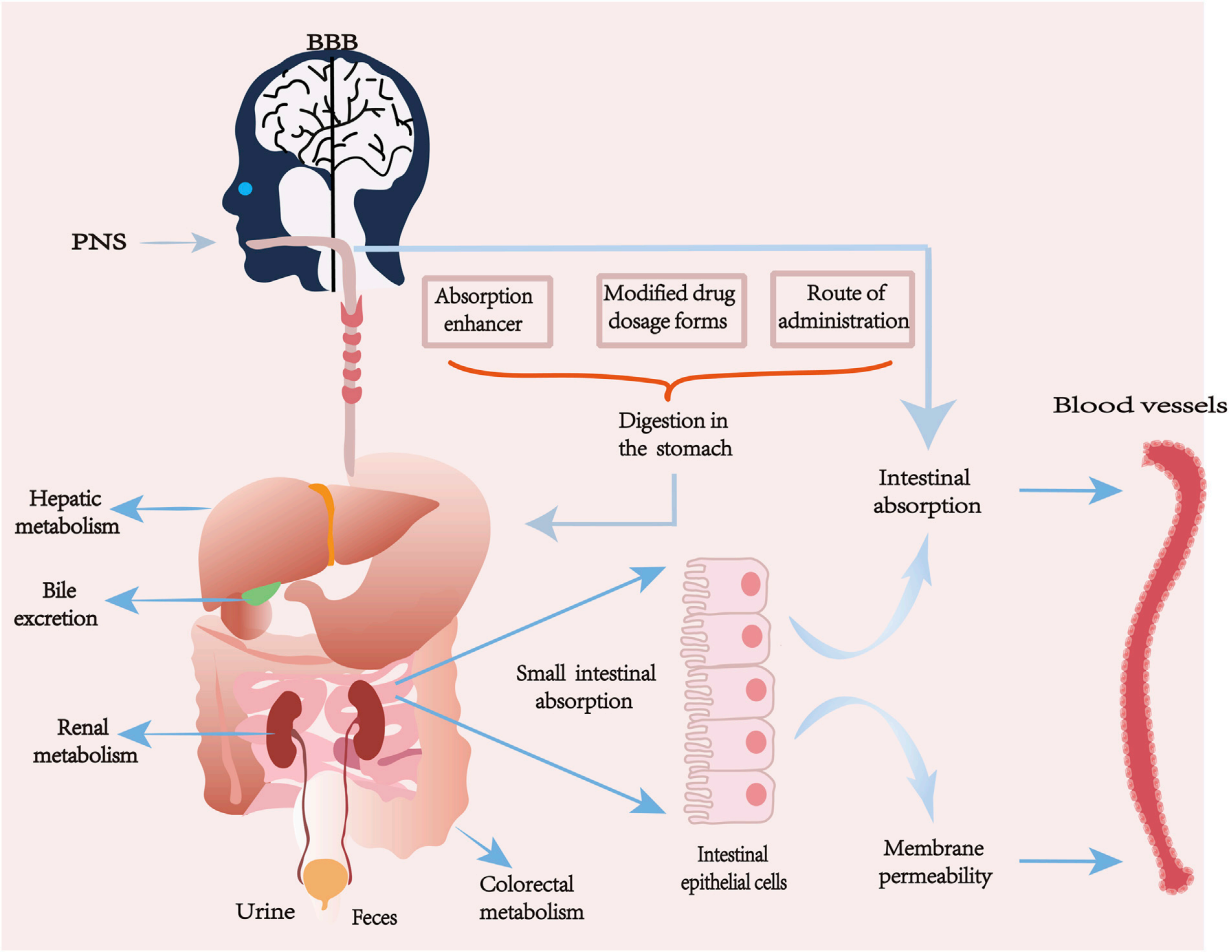
Metabolic pathways of ginsenoside PNS.

The pharmacokinetics of PNS is complex, with low absorption and bioavailability. To improve the related efficacy of PNS, it is necessary to conduct in-depth research on formulation selection, administration routes, and absorption mechanisms. Continuous exploration of novel drug delivery systems is required to address the quality control and dosage standardization issues of PNS formulations, ensuring that their clinical efficacy is reproducible and assessable, and increasing their bioavailability (see Figure 4; Table 1).

**TABLE 1:** Comparison of formulation strategies and routes of administration.

Administration route/formulation strategy	Description and mechanism	Main advantages	Limit
Traditional oral preparations	Tablets, capsules, etc. Absorption is limited by intestinal permeability	The patient compliance is high and it is easy to use	Bioavailability is extremely low, and there is a large individual variability
Oral novel drug delivery system	Biological adhesive patches, nanoemulsions, liposomes, etc. Improve absorption by enhancing mucosal retention, inhibiting efflux pumps, or opening tight junctions	Significantly improve bioavailability	The formulation process is complicated and the cost is high
Combined use of oral absorption promoters	Used in conjunction with BO or aspirin. BO can increase membrane fluidity, while aspirin can temporarily expand intercellular space	Simple and effective	May cause gastrointestinal irritation, long-term safety needs to be assessed
Injection administration	Xueshuantong injection. The drug is directly injected into the systemic circulation	Bioavailability is 100%, rapid onset of action, suitable for emergencies	Invasive procedures may cause allergic reactions
Nasal administration	The drug enters the brain directly through the olfactory nerve pathway	Avoid the BBB and first-pass effect to enhance brain targeting	Nasal mucosal irritation, limited dosage volume
Freeze-drying process	Applied to injectables to improve stability	Maintain ingredient activity, making it easy to store and transport	Mainly address stability, indirectly improving PK in the body

## 4 Pharmacological effects and mechanisms of PNS

PNS has multiple pharmacological activities, including anti-inflammatory, antioxidant, anti-apoptotic, anti-tumor, immunomodulation, improvement of microcirculation, and promotion of nerve regeneration.

### 4.1 Anti-inflammatory

PNS-like components have good anti-inflammatory effects, mainly exerting neuroprotective effects by modulating inflammatory signaling pathways, inhibiting microglia/astrocyte over-activation, and reducing the release of pro-inflammatory factors. Studies have shown that the inflammatory response of the central nervous system is closely linked to the activation of glial cells. Different types of saponins inhibit inflammatory mediators and cytokines from multiple pathways and attenuate the resulting waterfall cascade effect (Ruan et al., 2020).

PNS inhibits lipopolysaccharide (LPS) and interferon-gamma-induced microglial cell activation, reduces serum production of tumor necrosis factor- α (TNF- α ), IL-6, and IL-1β and NO release, inhibit the expression levels of p-IKK, p-IκB and p-p65 proteins, inhibit the activation of the NF-kappa-B (NF-κB) signaling pathway, and thus inhibit the occurrence of cellular inflammatory responses (Beamer and Shepherd, 2012; Wang Y. et al., 2024). PNS, by decreasing the expression levels of the pro-inflammatory factors IL-1β and TNF- α while increasing the expression of the anti-inflammatory factor IL-10, which in turn exerts an anti-inflammatory effect (Shi et al., 2017). The NF-κB signaling pathway and MAPK/ERK signaling are closely linked to the anti-inflammatory effect, and R1 can ameliorate amyloid β-induced inflammation by inhibiting SphK1-mediated NF-κB activation in PC12 cells, and it can also activate the peroxisome proliferator-activated receptor γ (PPARγ) to inhibit oxidized low-density lipoprotein-induced inflammatory cytokine production (Su et al., 2016; Zhang et al., 2019; Wang Y. et al., 2024). R1 was able to attenuate the damage of mouse hippocampal neurons (HT22 cells) by two mechanisms: on the one hand, activating the Akt/Nrf2 signaling pathway on the one hand, and inhibiting the activation of NLR family pyrin domain containing 3 (NLRP3) inflammatory vesicles on the other (Zhai et al., 2018).

The anti-inflammatory mechanism exerted by PNS is to play a regulatory role on inflammatory cells and inflammatory mediators through multi-target and multi-pathway synergism, which can be used to start from the classical inflammatory pathways, such as NF-κB, and the modulation of immune cell polarization and function, and to deeply study the potential therapeutic value of these properties in cardiovascular and cerebrovascular diseases, neurodegenerative diseases, and autoimmune diseases.

### 4.2 Antioxidant

Oxidative stress is a series of reactions caused by the accumulation of oxygen free radicals in the body due to the dysregulation of the body’s production and scavenging of oxygen free radicals caused by various external stimuli. Associated with increased production of reactive oxygen species and decreased effectiveness of the antioxidant system (Sies et al., 2017), oxidative stress leads to aberrant signaling in cells thus leading to a range of pathological responses. PNS can further play a role in protecting cerebrovascular and neuronal cells through multiple mechanisms such as direct scavenging of free radicals, enhancement of antioxidant enzyme activity, activation of the Nrf2 pathway, and protection of mitochondrial function.

PNS has a certain antioxidant activity capacity (Xiang et al., 2020), and is able to protect astrocytes from H_2_O_2_-induced injury by activating the antioxidant system, as well as alleviating the damage in SH-SY5Y cells under oxygen glucose deprivation/reoxygenation (OGD/R) conditions. In addition, it can help the brain resist damage caused by oxidative stress in neurological diseases by activating the PI3K/Akt/Nrf2 antioxidant signaling pathway (Zhou N. et al., 2014; Hu et al., 2018). When intracellular Ca^2+^concentration is elevated, energy metabolism is reduced, which catalyzes the activation of hypoxanthine production, PNS can inhibit the elevation of hypoxanthine, inhibit the production of free radicals, and also reduce the production of reductive coenzyme II-NADPH oxidase (Meng et al., 2014); on superoxide dismutase (SOD), enhance glutathione peroxidase (GSH-PX) activity and reducing malondialdehyde (MDA) and nitric oxide levels in brain tissue as a means of scavenging free radicals. The protective properties could also be enhanced by increasing the activities of antioxidant enzymes, which significantly increased the activities of antioxidant enzymes (including SOD, GSH-PX, and CAT) in SAMP8 mice and B16 melanoma cells by activating the nuclear factor erythroid 2-related factor 2 (Nrf2)/heme oxygenase-1 (HO-1) signaling pathway, and simultaneously decreased the oxidative damage markers, 8-hydroxylated deoxyguanosine (8-OHdG) levels.

In addition, the cellular antioxidant defense system was further enhanced by up-regulating the expression of mitochondrial uncoupling proteins 4 and 5, which effectively protected the properties of neuronal cells (Huang J. L. et al., 2017; Peiran et al., 2017). PNS inhibited endoplasmic reticulum stress in PC12 cells by inducing the thioredoxin-1 (Trx-1) response to the H_2_O_2_-induced oxidative damage exhibited a strong protective effect (Zeng et al., 2015).

### 4.3 Anti-apoptosis

Apoptosis is a cell death process caused by various factors inside and outside the body triggering the pre-existing death program in the cell, which has an important physiological regulatory mechanism in regulating the development of the organism, cell renewal and differentiation. Apoptosis occurs in addition to necrosis during ischemia, hypoxia, and ischemia-reperfusion, mainly through endogenous and exogenous pathways.

PNS inhibits aberrant apoptosis and autophagy in hippocampal neurons of learning- and memory-impaired mice, which is associated with reactivation of phosphatidylinositol 3-kinase/Akt/mammalian target of rapamycin signaling. It has been found that Rb1 possesses anti-apoptotic properties and reduces oxidative stress damage (Kim et al., 2012), When investigating the protective effect of Rb1 on PC12 cells, it was found that Rb1 was effective in increasing cell viability, exerting neuroprotection and avoiding apoptosis in the presence of H_2_O_2_ induced neurotoxicity and leading to cell death (Zeng et al., 2015). In addition, PNS can reduce neuronal apoptosis after cerebral hemorrhage in mice by inhibiting the JNK signaling pathway and down-regulating the protein expression levels of mitochondrial cytochrome C, Caspase-9 and Caspase-3 in the brain tissues (Li et al., 2009). The Caspase family and the Bal-2 family of proteins, as apoptosis research The main core object of apoptosis research, in most cases neuronal apoptosis occurs mainly through the endogenous pathway. PNS can reduce the expression of the key protease caspase-3 by inhibiting the transcription of caspase-3 mRNA and the cleavage activation of caspase-3 p20 protein in rat cerebral hemorrhage foci and peri-focal area and thus reduce the occurrence of neuronal apoptosis in the ischemia-reperfusion period.

Brain-derived neurotrophic factor (BDNF) can inhibit apoptosis by regulating the expression of Bal-2 and Bax proteins, etc. PNS can promote the expression of BDNF, which enhances the endogenous repair mechanism after cerebral ischemia, and helps to improve the neurological deficits in rats, reduce the scope of cerebral infarction, and reduce neuronal apoptosis. In addition, PNS also promotes the transcription of Bcl-2 mRNA and the expression of Bcl-2 protein in cerebral hemorrhage foci and around foci. It reduces neuronal apoptosis by enhancing the expression of anti-apoptotic genes. PNS exerts anti-apoptotic effects by reducing the occurrence of necrotic apoptosis in OGD/R brain microvascular endothelial cells through inhibition of the RIP1-RIP3-MLK signaling pathway and attenuating mitochondrial damage (Hu et al., 2022).

### 4.4 Anti-tumor

PNS exhibits certain inhibitory activity against various types of tumors. In the field of the central nervous system, neurological tumors are one of the most lethal forms of cancer in the United States. Gliomas account for approximately one-third of all tumors affecting the central nervous system and brain, with glioblastoma multiforme (GBM) being the most common primary malignant tumor in the central nervous system (Miller et al., 2021; Berger et al., 2022). PNS can inhibit the protein expression of p-AKT and p-mTOR in U251 and U87 cells, further inhibiting the PI3K/Akt/mTOR signaling pathway to regulate GBM proliferation, migration, and apoptosis (Sami and Karsy, 2013). PNS Rh2 can induce cancer cells to revert to non-cancerous cells, induce apoptosis in various tumor cells such as mouse glioma C6Bu-1 cells, and inhibit the growth and differentiation of mouse melanoma B16 cells.

In general, PNS has clear broad-spectrum antitumor effects. PNS exerts its antitumor effects by directly inhibiting the proliferation and migration of tumor cells, and can also prevent the deterioration of pathological processes by promoting cell apoptosis. In addition, it can be used as a sensitizer in combination with traditional antitumor drugs to reverse tumor cell resistance.

### 4.5 Immunomodulation

PNS regulates the expression of cellular immune inflammatory factors through multi-targets and multi-pathways, and has the effect of reducing oxidative stress and alleviating inflammation. In addition to PNS, flavonoids and other substances in Panax notoginseng have a deep impact on immune regulation at multiple levels and play important roles in various types of inflammation (Yang C. et al., 2024).

After gavage of purified Panax notoginseng root saponin and leaf saponin for 7 consecutive days, PNS was able to increase the rate of erythrocyte complement C3b receptor flowering, suggesting that it has the effect of enhancing the immune function of erythrocytes in the body. PNS can regulate the level of cytokine secretion, optimize the distribution of peripheral blood T-lymphocyte subpopulations, and promote the secretion of cytokines (e.g., IL-2) that have a protective role, and at the same time, inhibit the production of harmful cytokines, so as to play an immunomodulatory role. PNS also showed a slight hemolytic activity against mouse ovalbumin-specific IgG2b antibody in ovalbumin-induced immunized mice, and had a significant adjuvant effect on ovalbumin-specific antibody and cellular responses in mice (Qin et al., 2006). PNS has a regulatory role in immunomodulation through multiple pathways on inflammatory cells and mediators, and is important for immunomodulation. PNS exerts regulatory effects on inflammatory cells and mediators through various pathways, and has an important impact on immunomodulation.

### 4.6 Improvement of microcirculation

PNS can significantly improve microcirculation of brain tissue, its main mechanism of action is to increase blood flow to the brain by dilating blood vessels, thus improving blood supply to brain tissue; at the same time, it can also inhibit platelet aggregation, reduce the possibility of thrombosis, further improve microcirculation; regulate the function of the vascular endothelium, and reduce the damage of vascular endothelium, which is a good way to improve the effects on a series of cerebral vascular diseases, such as ischemia and hypoxia. It has a good improvement effect on a series of cerebrovascular diseases such as ischemia.

#### 4.6.1 Regulation of vascular endothelial function

Vascular endothelial growth factor (VEGF) is a protein that binds specifically to vascular endothelial cells and has the ability to promote the proliferation and migration of vascular endothelial cells, increase vascular permeability, and promote angiogenesis. It was found that Rg1 could regulate VEGF secretion from human umbilical vein endothelial cells through activation of PI3K/Akt and β-catenin/T-cell factor signaling pathways, thus protecting the vascular endothelium (Leung et al., 2006). When vascular endothelial cells are damaged, endothelial cell dysfunction occurs, which seriously affects the physiological and pathological processes such as vascular tone regulation, hemostasis and thrombosis, and vascular chronic inflammation, etc. PNS is able to regulate endothelial cell dysfunction in multiple ways, and it has a significant vascular endothelial protective effect. PI3K/Akt signaling pathway, inhibit Protein kinase C, and protect endothelial cell function (Lan et al., 2011). Rg1 can inhibit the mitochondrial apoptotic cascade, downregulate the expression of HIF1 α , activate the ERK signaling pathway, and resist the apoptosis of human umbilical vein endothelial cell fusion (EA.hy926) induced by Aβ25-35, and play a role in protecting the vascular endothelium (Yan et al., 2013).

PNS also has a role in promoting angiogenesis, which is the stimulation of endothelial cells to form new blood vessels, and is potentially therapeutic for cerebrovascular diseases such as chronic stroke (Giacca and Zacchigna, 2012). Can be shown to be pro-angiogenic by activating the VEGF-KDR/Flk-1 and PI3K-Akt-eNOS signaling pathways *in vivo* and *in vitro* (Yang B. R. et al., 2016). R1 also promotes the proliferation, migration, and tube formation of human umbilical vein endothelial cells, and activates the Ang2/Tie2 signaling pathway to promote angiogenesis (Zhong et al., 2020). By regulating the function of vascular endothelial cells, PNS are able to reduce the damage of vascular endothelial cells and maintain the integrity of blood vessels.

#### 4.6.2 Hemostasis and antithrombosis

PNS exhibits a highly dependent bidirectional regulatory effect on platelet function (promoting and inhibiting platelet aggregation), capable of exerting hemostatic and anti-thrombotic effects. PNS has the effect of promoting platelet aggregation, which can enhance platelet aggregation in a concentration-dependent manner. Among the saponins that show platelet aggregation effects, PNSFt1 is the most effective; Ft1 enhances platelet aggregation by activating the P2Y_12_ receptor-mediated signaling network, showing a dose-dependent synergistic effect with adenosine diphosphate (ADP). The rate of platelet aggregation increases with the dosage of Ft1, further inhibiting the production of cAMP, activating the phosphorylation of downstream PI3K and Akt in rat platelets through the P2Y12 signaling pathway, thus promoting platelet aggregation, making it the most effective coagulant *in vitro* (Gao et al., 2014). Ft1 can also enhance the thrombin-induced PLCγ2-IP3/DAG-[Ca2+]/PKC-TXA2 signaling pathway, promoting platelet aggregation to exert hemostatic effects (Liu Y. et al., 2019).

PNS also has the effect of inhibiting platelet aggregation, significantly suppressing collagen and ADP-induced platelet aggregation, inhibiting the reduction of platelets and the increase of fibrin degradation products, free calcium, TXA2, etc., inhibiting the thrombin-induced conversion from fibrinogen to fibrin, activating urokinase, promoting the dissolution of fibrin, improving blood flow, thus playing a role in anti-thrombosis (Wu et al., 2023). Pharmacological studies have found that PNS can inhibit thrombin-induced platelet aggregation by activating the PPAR-γ pathway and its downstream PI3K/Akt/eNOS pathway, effectively improving the hypercoagulable state in the body to reduce thrombosis (Shen et al., 2017). Rg1 can inhibit abnormal platelet activation by suppressing the ERK signaling pathway, thereby reducing arterial thrombosis induced by Fe Cl3 in mice (Zhou Q. et al., 2014). In a study on a rat model of cerebral ischemia with thrombosis in multiple sites such as cerebral artery occlusion, after PNS intervention, the levels of free fatty acids (FFA), adenosine triphosphate, phosphocreatine, and Ca^2+^ in rat brain tissue were significantly downregulated, and the area of cerebral ischemic infarction was significantly reduced, indicating that PNS has a good effect in alleviating cerebral thrombosis (Wu et al., 2022).

PNS’s hemostatic and antithrombotic effects on platelets are bidirectional, reflecting its multi-component and multi-target characteristics in different contexts. However, the antiplatelet and profibrinolytic effects of PNS may have additive effects with aspirin, clopidogrel, warfarin, or novel oral anticoagulants, increasing the risk of bleeding, allergies, gastrointestinal reactions, and other adverse responses. Analysis using a fixed-effects model for the occurrence of hemorrhagic transformation indicated that there was no significant difference in the incidence of hemorrhagic transformation between the groups (RR: 0.62; 95% CI: 0.34 to 1.14; *p* = 0.13, I^2^ = 0%) (Liu Y. et al., 2024). Based on the current high-level evidence, for patients with clear indications, the overall therapeutic net benefit of PNS outweighs its manageable risks. Its use should be guided by evidence of its anticoagulant and circulation-improving effects in pathological conditions, with full awareness of the potential risks when used in combination with standard anticoagulant therapy, to achieve safe and effective rational medication.

### 4.7 Promoting nerve regeneration

PNS plays an important role in the regulation of the nervous system, and its main components have the ability to protect neuroactivity, excite the brain center, promote blood circulation in the brain, enhance memory, improve cognitive impairment, and regulate neurotransmitters to promote nerve regeneration (Liu S. Z. et al., 2019).

As a BDNF, PNS can increase the expression of BDNF to upregulate Akt/CREB to restore the mechanism of neurological function and promote neurogenesis and oligodendrogliogenesis after ischemic stroke (Zhu et al., 2021a). As a potential novel neuroprotective agent, it can reduce neurological deficits after traumatic brain injury (TBI), ameliorate neuronal apoptosis and promote neuronal regeneration in TBI by inhibiting the ERK signaling pathway (Pei et al., 2023). PNS also upregulates nerve growth factor (NGF) and BDNF in spinal cord transected rats, providing neuroprotection, promoting axon growth, and improve hindlimb motor function (Wang et al., 2015). It can also attenuate bupivacaine-induced neurotoxicity by activating the Jak1/Stat3 pathway and triggering the upregulation of Mcl1 to rescue apoptosis and promote neuronal survival; and attenuate hepta flurane-induced neuronal cell damage by modulating the AKT signaling pathway to protect the neuronal cells and promote nerve regeneration (Yang et al., 2017; Yang et al., 2024c).

## 5 Effects on cerebrovascular system

**FIGURE 5:**
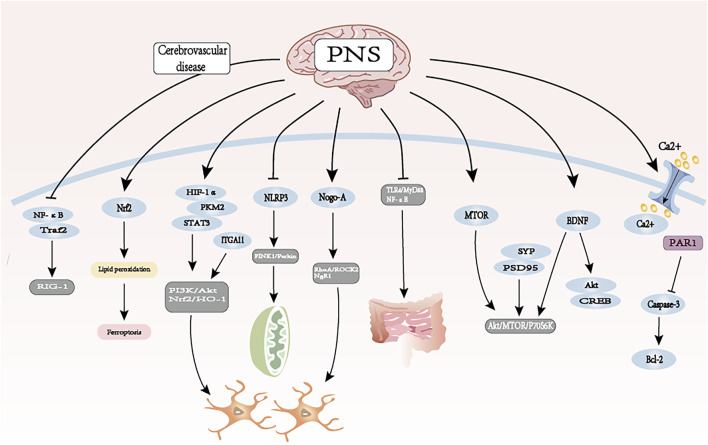
The therapeutic mechanism and critical pathways of PNS against cerebrovascular diseases.

PNS has effects on the cerebrovascular system, such as increasing cerebrovascular blood volume, anti-atherosclerosis, decreasing platelet surface activity, inhibiting platelet activation, adhesion and aggregation, promoting vascular neovascularization and repair, and anti-thrombosis. It can play a role in protecting the cerebrovascular system by improving cerebral ischemia-reperfusion injury, decreasing the permeability of blood-brain barrier, improving the function of cerebral blood flow, promoting neural remodeling and synaptic reconstruction, and improving the neurological deficits (see Figure 5; Table 2).

**TABLE 2:** Summary of the mechanisms and effects of panax notoginseng saponins on cerebrovascular diseases.

Disease	Experimental model	Dose	Mechanisms	Effects	References
IS	*In vivo:* SD rats	*In vivo*: 20 mg/kg (i.p. injection)	*In vivo:* aspartate, phospholipids, NAA, ATP, GSH↑ ROS, TUNEL-positive neurons↓	Reduces infarct size and ameliorates neurological deficits by ameliorating neuronal damage and inhibiting glial cell activation in MCAO/R rats	Zhu et al. (2020)
IS	*In vivo:* SD rats	*In vivo:* 7.2 mg/100g (i.p. injection)	*In vivo*: NgR1, RhoA, ROCK2 ↓	By inhibiting the overexpression of NgR1, RhoA, and ROCK2, neuroprotective effects were provided in a rat model of cerebral ischemia and in SH-SY5Y cells exposed to oxygen/glucose deprivation injury	Shi et al. (2016)
	*In vitro:* SH-SY5Y cells	*In vitro:* 160 μg/mL	*In vitro*: NgR1, RhoA, ROCK2 mRNA↓		
IS	*In vivo:* SD rats	*In vivo:* 40,80,160 mg/kg (i.p. injection)	*In vivo*: LC3-II, P62↑ NLRP3, caspase-1, IL-1β, IL-18↓	Promotes mitophagy via the PINK1/Parkin pathway, inhibit NLRP3 inflammasome activation, and reduce cerebral I/R injury	Xiao et al. (2022)
IS	*In vivo:* SD rats	*In vivo*: 16.5 mg/kg (i.p. injection)	*In vivo*: RIG-I, TRAF2, NF-κB (p65)TNF-α, IL-8↓	Activation of the RIG-I signaling pathway inhibits the related signaling molecules TRAF2 and NF-κB, thereby exerting the anti-inflammatory properties of PNS in cerebral ischemia	Zhang et al. (2021)
IS	*In vitro:* C17.2 cells	*In vitro*: 25, 50, 100, 200 μg/mL	*In vitro:* p-Akt, p-mTOR, p-p70S6K, BDNF, SYP, PSD95↑	Improved the survival of C17.2 cells after OGD/R injuries and promoted C17.2 cells to differentiate into neurons and astrocytes, enhanced the synaptic development and plasticity of C17.2 cells	Gao et al. (2024)
IS	*In vivo:* SD rats	*In vivo:* 20, 40 mg/kg (i.p. injection)	*In vivo*: glucose, GLUT 1/3, MCT1, CS, lactate transportation, ATP↑	Increases cerebral glucose and lactate transportation and ATP levels, ameliorates neuronal mitochondrial function after IS	Liu et al. (2022)
	*In vitro:* Neuro 2a	*In vitro:* 5, 10, 20, 100, 200 μg/mL	*In vitro*: GLUT 1/3, MCT1, CS, ATP↑		
IS	*In vivo:* SD rats	*In vivo:* 30, 60 mg/kg (i.v. injection)	*In vivo*: neurological deficit scores, cerebral infarct volumes ↓	Reducing neurological scores and infarct volume in MCAO rats demonstrates effective neuroprotective effects	Lin et al. (2015)
IS	*In vivo:* SD rats	*In vivo*: 10, 20, 40 mg/kg (i.p. injection)	*In vivo*: BDNF, NGF, NT-4↑ infarction volumes, neuronal loss↓	Upregulate Akt/CREB by increasing BDNF expression, promoting neurogenesis and oligodendrocyte generation	Zhu et al. (2021a)
IS	*In vitro:* Cortical neurons from rats	*In vitro*: 10 μmol/L	*In vitro:* Activation of p-PLCβ/p-PLCγ, IP3R1, Ca^2+^↓	Alleviating OGD/R-induced cellular damage by activating ER stress inhibition through PLC.	Wang et al. (2017)
IS	*In vivo:* SD rats	*In vivo:* 20 mg/kg (i.p. injection)	*In vivo:* Cerebral blood flow, VEGFR-2 expression↑	Alleviating OGD/R-induced cellular damage by activating ER stress inhibition through PLC.	Zhu et al. (2021b)
	*In vitro:* HBMEC cells	*In vitro:* 6.26–100 μM	*In vitro:*Protein expression levels of NAMPT↑		
HS	*In vivo:* C57BL/6J mice	*In vivo:* 50, 100, 200 mg/kg (intragastric administration)	*In vivo*: IL-1β, IL-6, Neurological deficits, mNSS scores ↓ TGF-β, IL-10↑	By enhancing lymphatic vessel formation and meningeal lymphatic drainage function, thereby alleviating ICH inflammation and promoting neurological recovery	Yu et al. (2024)
HS	*In vivo:* SD rats	*In vivo:* 12.5, 75, 200 mg/kg (i.p. injection)	*In vivo*: cerebral hematoma, K^+^ ↓ Na^+^↑		Nie et al. (2006)
SAH	*In vivo:* C57BL/6J mice *In vitro:* HT22 cells	*In vivo:* 20, 60, 100 mg/kg (i.p. injection) *In vitro:* 20, 60, 100 mM	*In vivo*: cell viability↑, cytotoxicity ↓ *In vitro:* LDH↓	Reduction of neuronal apoptosis and cerebral edema in experimental SAH models via the ITGA11 pathway	Hou et al. (2025)
Dementia	*In vivo:* Male ICR mice	*In vivo*: 6 mg/kg (Rg1-6,Rb1-6), 12 mg/kg (Rg1-12,Rb1-12) (i.p. injection)	*In vivo*: AChE, 5-HT↑	Rg1 was more effective than Rb1 in improving escape acquisition deficiency induced by scopolamine, and could significantly reduce the scopolamine-induced increase in AChE activity in the hippocampus in mice	Wang et al. (2010)

Refs., references; ATP, Adenosine triphosphate; ROS, Reactive Oxygen Species; APP, amyloid precursor protein; GSH, Glutathione; MCAO, Middle cerebral artery occlusion; IL-1β, Interleukin-1β; NLRP3, NOD-like receptor protein 3; TRAF2, TNF Receptor Associated Factor 2; TNF-α, Tumor necrosis factor-α; IS, ischemic stroke; BDNF,Brain-derived neurotrophic factor; NGF, Nerve growth factor; OGD/R, Oxygen glucose deprivation/reperfusion;NAMPT, Nicotinamide phosphoribosyltransferase; SAH, Subarachnoid hemorrhage; LDH, Lactate dehydrogenase; AChE, acetylcholinesterase; 5-HT, 5-hydroxytryptamine.

### 5.1 Anti-ischemic stroke (IS) effects

IS is an acute cerebrovascular disease in which cerebral blood flow is interrupted leading to necrosis of brain tissue, and the core mechanism is local cerebral ischemia and secondary injury (Hu et al., 2017). If cerebral ischemia is incompletely blocked and then returns to normal, cerebral ischemia reperfusion injury (CIRI) is triggered, and PNS has a significant neuroprotective effect on focal CIRI in rats (Yang P. F. et al., 2016; Li H. et al., 2018). PNS induces a variety of pharmacological effects in a multiscale mechanism of the pathophysiology of cerebral ischemia including anti-inflammatory activity, reduced oxidative stress, anti-apoptosis, inhibition of amino acid excitotoxicity, reduction of intracellular calcium overload, protection of mitochondria, repair of the blood-brain barrier and promotion of cell regeneration (Wang et al., 2021b). Compared with hemodialysis, ischemia-reperfusion injury is the most important part of ischemic stroke progression, and improvement of BBB injury is important to reduce ischemia-reperfusion injury (Feng et al., 2021a; Yang et al., 2022).

A systematic review and meta-analysis on the effect of PNS on acute ischemic stroke (AIS) indicates that treatment with Xuesaitong and PNS injection not only improves daily living activities, alleviated neurological deficits, improved cerebral blood flow, and attenuated CIRI, but also was well tolerated and reduced the incidence of adverse events (Wang et al., 2021a; Shi et al., 2023; Liu Y. et al., 2024). In a rat model of focal middle cerebral artery embolism (MCAO), PNS significantly ameliorated focal CIRI by decreasing the volume of cerebral infarction and inhibiting the release of the inflammatory factors IL-1β and TNF-α (Sun et al., 2020). PNS attenuated the extent of BBB destruction by cerebral ischemia/reperfusion (I/R) in the MCAO model, and reduced the extent of middle cerebral artery blockage in rats. Focal cerebral ischemia/reperfusion and reduce the water content of brain tissue and the area of cerebral infarction, and improve its neurobehavioral function and pathological characteristics (Luo et al., 2019; Dong et al., 2022). It can also reduce infarct area and improve neurological deficits by regulating brain small molecule metabolism to protect the brain from CIRI, ameliorating neuronal injury in middle cerebral artery embolization/reperfusion (MCAO/R) rats and inhibiting glial cell activation (Zhu et al., 2020).

Multiple saponins isolated from the aqueous extract of Panax quinquefolium exerted neuroprotective effects on damaged human neuroblastoma (SH-SY5Y) cells (Liu et al., 2018), Suppressing the overexpression of NgR1/RhoA/ROCK2 by modulating the myelin-associated inhibitory molecule, Nogo-A, *in vivo* and *in vitro* in the rat model of cerebral ischemia and in the rat model of exposure to OGD/R injury in the SH-SY5Y cell model providing neuroprotection (Shi et al., 2016). PNS also attenuates CIRI in rats by inhibiting the activation of NLRP3 inflammatory vesicles and promotes mitochondrial autophagy through the PINK1/Parkin pathway (Xiao et al., 2022). Neuronal energy failure during the acute phase of focal cerebral ischemia can also be circumvented by ameliorating mitochondrial impairment (Liu et al., 2022).

Inhibition of microglia activation and reduction of inflammatory response in the CNS is essential to reduce brain damage caused by IS as seen in the results of a PNS on microglia inflammatory response in cerebral ischemia (Duan et al., 2024). PNS can protect ischemically injured brain cells by activating the PI3K/Akt and Nrf2 signaling pathways, and it can also inhibit the downregulation of HIF-1α/PKM2/STAT3 signaling in microglial cells to reduce inflammation induced by microglial cell activation. PNS can also activate the RIG-I signaling pathway, and inhibit the related signaling molecules, TNF receptor associated factor 2 (TRAF2) and NF-κB, which are essential for the reduction of IS-induced brain injury. (NF-κB) exerts the anti-inflammatory properties of PNS in cerebral ischemia (Zhang et al., 2021; Gao J. et al., 2022). Activation of the Nrf2 signaling pathway attenuates inflammation, regulates the expression of iron overload and lipid peroxidation-related proteins and the activity of antioxidant enzymes as a means of inhibiting iron death and attenuating CIRI (Wang L. L. et al., 2024); it also inhibits the TLR4/MyD88/NF-κB signaling pathway, and inhibits cerebral ischemia through the microbiome-gut-brain axis. -gut-brain axis to inhibit the stimulation of astrocytes and microglia in the ischemic zone of the brain, restoring the structure of the BBB and attenuating CIRI (Zhang et al., 2024). In addition, PNS promotes the differentiation of cells expressing immature neuroblasts in the olfactory bulb after ischemia and reperfusion, and promotes neuronal regeneration (He et al., 2015). In another study, PNS stimulated C17.2 neural stem cell (NSC) differentiation and neuronal synapse development through mTOR signaling against OGD/R injury, demonstrating the neural differentiation-inducing properties of PNS in mouse C17.2 NSCs after OGD/R injury with the involvement of Akt/mTOR/p70S6K signaling pathway (Gao et al., 2024).

Rg1, as one of the major components of PNS, significantly reduces the volume of cerebral infarction and alleviates neurological dysfunction after I/R injury, and exhibits multiple mechanisms of physiological activity against cerebral ischemia and reperfusion injury. Antioxidant activity and related apoptosis through Akt, Nrf2/HO-1, PPARγ/HO-1, extracellular regulated protein kinase (ERK), p38 and c-Jun N-terminal kinase (JNK) pathway (or mitochondrial apoptosis pathway) and c-Caspase-3/ROCK1/MLC pathway; through MAPK pathway (JNK1/2 +), ERK1/2 and PPARγ/HO-1; and through MAPK pathway (JNK1/2 +), ERK1/2 and PPARγ/HO-1. ERK1/2 and PPARγ/HO-1 pathways), endoplasmic reticulum stress (ERS), anti-inflammatory and immunostimulation-associated activities of apoptosis or necrosis induced by high mobility group protein 1 (HMGB1) TLR2/4/9 and receptor for end products of advanced glycosylation (RAGE) pathways, and activation of NF-κB; Anti-inflammatory and immunostimulatory responses to apoptosis or necrosis; neuronal cell cycle, proliferation, differentiation, and regeneration; and regulation of energy metabolism and cellular ATP levels, BBB permeability, excitatory amino acids, and other processes, including NGF activation, excitotoxicity, and Ca^2+^ excess influx into neurons, which are mechanisms that play significant neuroprotective roles against brain ischemic injury (Lin et al., 2015; Xie et al., 2018b).

After CIRI, R1 promotes angiogenesis and improves energy metabolism mainly through antioxidant, anti-apoptotic and anti-inflammatory mechanisms (Tong et al., 2019). R1 intervenes in the degradation and redistribution of tight junctions through the Caveolin-1/MMP2/9 pathway and attenuates BBB permeability, cerebral infarct volume, and neurological impairments in acute cerebral ischemic rats (Liu et al., 2021). Increasing cerebral glucose and lactate transport and ATP levels and improving mitochondrial dysfunction improves cerebral energy metabolism to circumvent neuronal energy failure caused by acute ischemic stroke (Liu et al., 2022). R1 upregulates Akt/CREB by increasing the expression of BDNF, and restores neurological mechanisms through the participation of Akt/mTOR/p70S6K signaling pathway. R1 promotes oligodendrogenesis after ischemic stroke by increasing the expression of BDNF, thereby improving CIRI and long-term neurological recovery after ischemic stroke (Zhu et al., 2021a). R1 inhibits neuronal apoptosis and the expression of endoplasmic reticulum stress-associated pro-apoptotic proteins, promotes neural stem cell proliferation and differentiation, and protects neurons, endothelial cells, and astrocytes in ischemic stroke to mitigate brain injury. cells as a way to attenuate brain damage (Wang et al., 2017; Yang et al., 2020).

PNS has multi-scale pharmacological effects and good safety profile, which can effectively reduce the volume of cerebral infarction, improve neurological deficits and alleviate reperfusion injury. At the same time, clinical evidence supports that it has a good safety and tolerability in the treatment of AIS, which can improve the recovery of patients’ neurological function and serious adverse events are rare.

### 5.2 Anti-hemorrhagic stroke (HS) effects

HS is the most fatal form of stroke and mainly includes cerebral hemorrhage and subarachnoid hemorrhage (SAH) (Ohashi et al., 2023). The underlying cause of neurological impairment is the hematoma itself and the secondary damage around it. Brain edema, inflammatory response, apoptosis, ischemia of the tissues around the hematoma, and oxygen free radicals induced by cerebral hemorrhagic injury can enter the brain parenchyma through the damaged BBB and further exacerbate brain tissue damage (Chen et al., 2022).

PNS can significantly improve edema and hematoma in patients with ICH, and hematoma resorption and neurological function can be significantly improved in the treatment of ICH with Xueshuantong injections (175 mg/d) for 2 weeks (Gao et al., 2012). In an analysis, ICH patients treated with PNS demonstrated better outcomes than untreated ICH patients in a number of areas, including effective rate, neurological deficit score, intracranial hematoma volume, intracranial edema volume, and Barthel index (Xu et al., 2014). As the drainage system of the brain, the meningeal lymphatic system can also reduce hematoma volume, improve neurological inflammation, and enhance neurological recovery by enhancing lymphatic vessel formation and meningeal lymphatic drainage function (Yu et al., 2024). PNS significantly reduced neurological deficits in an experimental SAH model by inhibiting the expression level of ITGA11, activating the PI3K-Akt pathway to reduce neuronal apoptosis and cerebral edema, facilitating the absorption of intracranial hematomas after hemorrhage, improving the peripheral ischemic area blood flow, and decreasing the apoptosis of peripheral neurons, and thus exerting neuroprotective effects (Hou et al., 2025).

The formation of cerebral edema after cerebral hemorrhage is related to thrombin, and it is found that the expression of prothrombin receptor 1 (PAR1) in brain tissue after cerebral hemorrhage has a positive correlation with apoptosis and the emergence of cerebral edema, and thrombin gradually released from the hematoma may aggravate the cerebral injury by continuously activating PAR1 (Moller et al., 2000). PNS can prolong the clotting time of plasminogen and thrombin, and reduce the activation of platelet thrombosis. clotting time, reduce platelet activation, reduce apoptosis by inhibiting the transcription of Caspase-3 mRNA and the activation of Caspase-3 protein cleavage, and promote the transcription and protein expression of Bcl-2 gene to promote the survival of neurons and repair of damage in the brain after cerebral hemorrhage (Qureshi et al., 2003).

PNS may increase brain damage by up-regulating basic fibroblast growth factor (BFGF), which is the most important factor in the development of cerebral hemorrhage. PNS can promote neuronal survival and injury repair after cerebral hemorrhage by up-regulating the expression of BFGF, laminin, the transcription of anti-apoptotic gene Bcl-2 mRNA and the expression of Bcl-2 protein, up-regulating the expression of the rat forebrain excitatory amino acid receptor AMPA receptor subunit GluR2, the cerebral hemorrhage peripheral microtubule-associated protein-2, the growth-associated protein-43, and X-linked apoptosis inhibitory protein XIAP, as well as down-regulating the expression of the XIAP protein, downregulate the expression of rat forebrain excitatory amino acid receptors NR1, NR2A, NR2B, aquaporin-4, ICAM-1, and TNF- α , reduce the release of plasma MMP-9 and IL-6, inhibit the elevation of complement C3, and downregulate the content of fibrinogen and the time of fibrinogen coagulation to promote neuronal survival in the brain after cerebral hemorrhage, damage repair and neural remodeling.

PNS has shown significant neuroprotective and repair effects through multi-target mechanisms, including accelerating intracranial hematoma resorption, reducing cerebral edema, improving neurological deficits, and inhibiting secondary damage. PNS can effectively improve clinical outcomes and multiple functional indicators by enhancing meningeal lymphatic drainage, inhibiting inflammatory responses, regulating apoptosis-related pathways (such as inhibiting Caspase-3 and upregulating Bcl-2), protecting the integrity of the blood-brain barrier, and promoting nerve remodeling.

### 5.3 PNS and vascular dementia

VD is the most common form of dementia after neurodegenerative dementia. Oxidative stress, neuroinflammation, neurotransmitter system and mitochondrial dysfunction, lipid metabolism disorders, and changes in growth factors are all important factors that exacerbate the pathological process of dementia (Du et al., 2017).

Rg1 significantly reduces MDA, IL-β, TNF-α levels in the brain tissue of VD rats, and increases SOD activity in brain tissue, indicating that ginsenoside Rg1 has antioxidant and anti-inflammatory effects. It can reduce oxidative stress levels and inflammatory responses in the brain tissue of VD rats, potentially by inhibiting apoptosis of hippocampal neurons in VD rats through the activation of the MEK5/ERK5 signaling pathway, and improving inflammatory and oxidative damage in hippocampal tissue. Rg1 and Rb1 can increase ACh levels in the hippocampus and both can inhibit the reduction of 5-hydroxytryptamine (5-HT) induced by scopolamine, thereby improving dementia, enhancing memory, and exerting cognitive-enhancing effects (Wang et al., 2010). By regulating the Notch signaling pathway to activate the nicotinamide phosphoribosyltransferase-nicotinamide adenine dinucleotide-Sirtuin 1 (NAMPT-NAD+-SIRT1) cascade reaction, this approach promotes angiogenesis following stroke-induced ischemia, thereby improving vascular cognitive impairment and dementia (Zhu et al., 2021b).

PNS significantly improves cognitive function through antioxidant, anti-apoptotic, cholinergic nervous system regulation, and neuroplasticity regulation, demonstrating significant anti-dementia effects and providing new avenues for the treatment of diseases accompanied by neurological dysfunction (Wang et al., 2010).

## 6 Effects of PNS on neurological disorders

**FIGURE 6:**
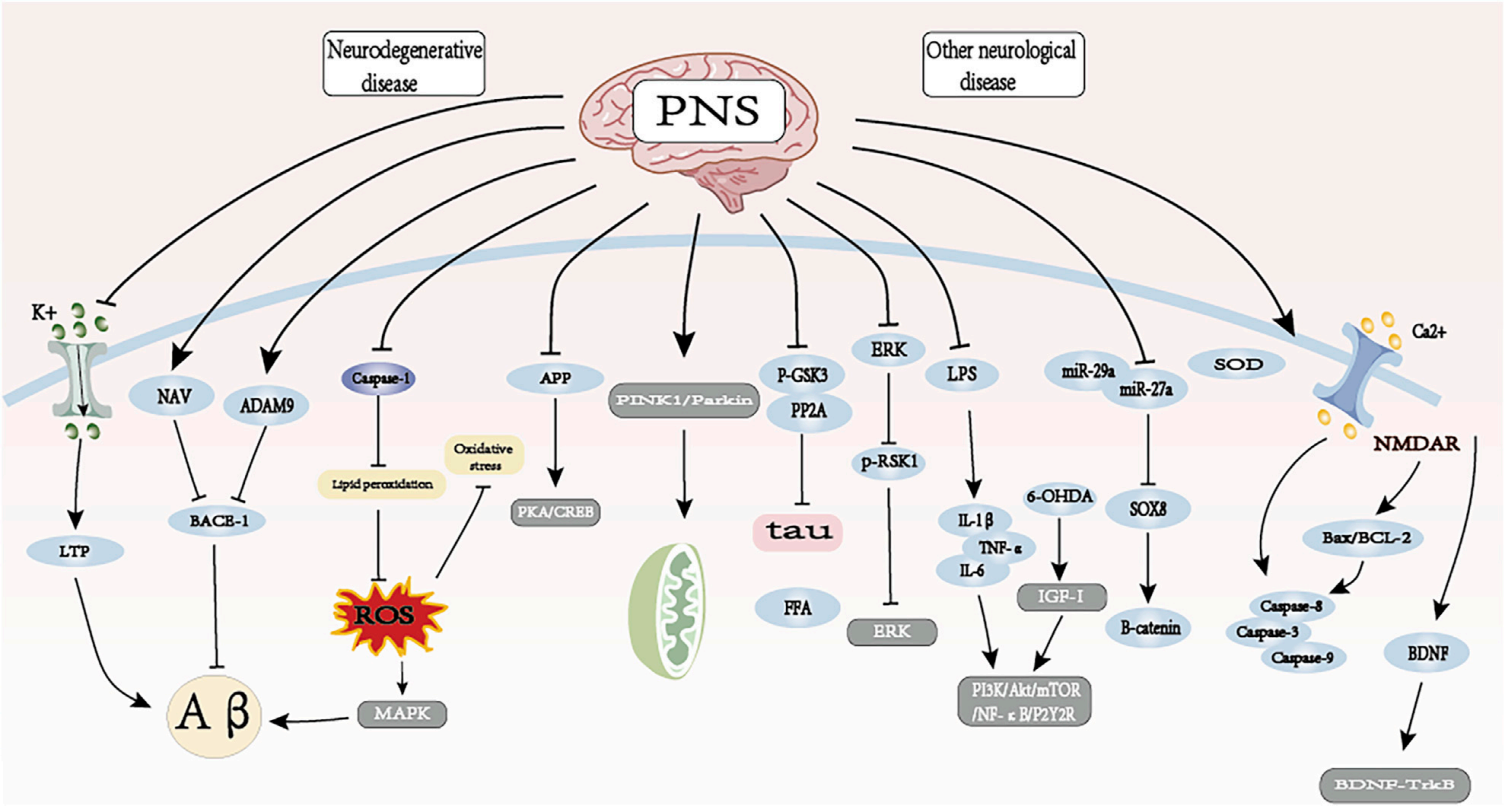
The therapeutic mechanism and critical pathways of PNS against neurological disorders.

Modern pharmacological studies have found that PNS has good preventive and therapeutic effects on cerebral nervous system diseases such as AD, Parkinson’s disease, depression, and other neurological diseases (Su et al., 2014). It can inhibit neuronal apoptosis through antioxidant, anti-apoptotic, and anti-neuroinflammatory activities, promote synaptic plasticity and nerve regeneration, and protect damaged neurons, which can slow down and prevent the occurrence of neurological disorders, and it is an effective and safe natural drug for the treatment of neurological diseases (Qu et al., 2020) (see Figure 6; Table 3).

**TABLE 3:** Summary of the mechanisms and effects of panax notoginseng saponins on neurological disorders.

Disease	Experimental model	Dose	Mechanisms	Effects	References
AD	*In vivo:* C57BL/6J mice	*In vivo*: 5 mg/kg (gavage)	*In vivo:* Membrane excitability of hippocampal neurons↑ AP threshold↓	Increased the membrane excitability of CA1 pyramidal neurons in hippocampal slices by lowering the spike threshold possibly through a mechanism involving in the inhibition of voltage-gated K^+^ currents	Yan et al. (2014)
AD	*In vivo:* Tg mice	*In vivo:* 10 mg/kg (i.p. injection)	*In vivo*: γ secretase activity↓	Induces neuroprotection through ameliorating amyloid pathology, modulating APP process, improving cognition, and activating PKA/CREB signaling	Fang et al. (2012)
	*In vitro:* Rat neuroblastoma B103 cells	*In vitro:* 5, 10 μM	*In vitro*: PKA activity, CREB phosphorylation↑		
AD	*In vitro:* PC12 cells	*In vitro:* 1, 5, 10, 50, 100 μM	*In vitro*: cell viability, p-ERK, p-JNK, p-p38 ↑ ROS production, mitochondrial damage↓	Protects PC12 neuronal cells from Aβ25–35-induced neurotoxicity by inhibiting oxidative stress, apoptosis, and stress-activated MAPK signaling pathways	Ma et al. (2014)
AD	*In vivo:* C57BL/6J mice	*In vivo*: 5 mg/kg (gavage)	*In vivo*: MWM test behavior ↑ expression of BACE1, excessive cleavage of Navβ2↓	Aβ1-42-induced injured neurons were saved by increasing cell viability	Hu et al. (2020)
	*In vitro:* Cortical or hippocampal cells	*In vitro:* 5 μM	*In vitro*: cell viability↑ expression of BACE1, excessive cleavage of Navβ2↓		
AD	*In vivo:* SAMP8 mice	*In vivo*: 50, 100 mg/kg (gavage)	*In vivo*: SOD activity, GSH-Px activity,GSH, 8-OHDG, MDA↑	Improve learning and memory in SAMP8 mice with dementia by enhancing mitochondrial autophagy associated with the PINK1/parkin pathway and promoting the recruitment of Parkin	Yang et al. (2024b)
PD	*In vivo:* C57BL/6J mice	*In vivo*: 20, 50, 100 mg/kg (gavage)	*In vivo:* expression of iNOS, Iba-1, PI3K, AKT, NFκB, and IκB ↓	Inhibit excessive microglial activation in PD models by reducing microglia-mediated inflammation	Wu et al. (2025)
	*In vitro:* BV-2 cell	*In vitro:* 50, 100, 200, μg/mL	*In vitro*: ↑ Iba-1, NO, TNF-α, IL-6, IL-1β, iNOS,COX2↓		
PD	*In vivo:* female Wistar rats	*In vivo:* 10 mg/kg (i.p. injection)	*In vivo*: protein and gene expression of Bcl-2 and JB-1, gene expressions of TH and DAT↓	Rg1 has neuroprotective effects on the nigrostriatal system in this model of PD induced by 6-OHDA, mediated by the IGF-IR signaling pathway	Xu et al. (2009)
	*In vivo:* anti-rat DAT, anti-rat Bcl-2	*In vivo:* 10 mg/kg (i.p. injection)			
PD	*In vitro: C. elegans* N2	*In vitro:* 0.1, 0.5, 1, 5, 10 μM	*In vitro*: lifespan, sod-3, cat-2↑ egl-1/BH-3, ced-3/caspase-9↓	Rescuing DAergic neurodegeneration in the 6-OHDA-induced *C. elegans* model through suppressing apoptosis mediators and stimulating antioxidant enzymes	Chalorak et al. (2021)
PD	*In vivo:* Kunming mice	*In vivo*: 100 mg/kg (i.p. injection)	*In vivo*: Trx-1, COX-2↑ TH expression↓	Provided neuroprotection against the loss of dopaminergic neurons and behavioral impairment caused by MPTP.	Luo et al. (2011)
Depression	*In vivo:* NIH mice	*In vivo*: 50, 100 mg/kg (gavage)	*In vivo*: NA↑ ACTH, corticosterone↓	Rg3 exerts an antidepressant-like effect by modulating monoamine neurotransmitters	Zhang et al. (2016)
Depression	*In vivo:*Wistar rats	*In vivo*: 50, 100 mg/kg (oral)	*In vivo*: Bcl-2, expression of BDNF, PI3K, Akt, p65 NF-κB ↑ IL-6, IL-1β, TNF-α↓	Improves symptoms of depression by activating PI3K/Akt/NF-κB pathway	Zhan et al. (2022)
Depression	*In vivo:* ICR mice	*In vivo:* 20, 40 mg/kg (gavage)	*In vivo:* IDO expression, KYN/TRP ratio↑ IL-6, TNF-α↓	Rg3 was effective in ameliorating depressive-like behavior induced by immune activation	Kang et al. (2017)
Depression	*In vivo:* SD rats	*In vivo:* 20, 40 mg/kg (gavage)	*In vivo*: Tau,GABA↑ Glu, Asp↓	Improves depressive symptoms by modulating hippocampal amino acid levels in the long-term chronic stress response and preventing neurotoxicity of excitatory amino acids	Wu et al. (2012)
TBI	*In vivo:* SD rats	*In vivo:* 20, 40, 80 mg/kg (i.p. injection)	*In vivo*: Bcl-2↑, brain edema, Bax, caspase-3, IL-6, IL-1, TNF-α↓	Improve neuronal apoptosis in brain injury by inhibiting the ERK signaling pathway	Pei et al. (2023)
		*In vivo:* Male ICR mice	*In vivo*: 6 mg/kg (Rg1-6,Rb1-6), 12 mg/kg (Rg1-12,Rb1-12) (i.p. injection)	*In vivo*: AChE, 5-HT↑	

SOD, superoxide dismutase; CREB, Cyclic-AMP response binding protein; ROS, Reactive Oxygen Species; BACE1, β-site amyloid cleavage enzyme 1; NaV, Voltage-gated Na+ channels; MDA, Malondialdehyde; 8-OHdG, 8-hydroxy-2 deoxyguanosine; GSH-PX, Glutathione peroxidase; BACE1, β-site APP cleaving enzyme 1; NO, nitric oxide; Bcl-2, B-cell lymphoma-2; MAPK, mitogen-activated protein kinase; COX2, cyclooxygenase 2; Akt, protein kinase B; TNF-α, Tumor necrosis factor-α; IL-1β, Interleukin-1β; IL-6, Interleukin-6; MPTP, 1-methyl-4-phenyl-1,2,3,6-tetrahydropyridine hydrochloride; Trx-1, thioredoxin-1; BDNF, brain-derived neurotrophic factor; DAT, dopamine transporter; 6-OHDA, 6-Hydroxydopamine; GABA, Gama-aminobutyric acid; Glu, Glutamate; Bax, Bcl-2-associated X protein; AChE, acetylcholinesterase; 5-HT, 5-hydroxytryptamine.

### 6.1 PNS and Alzheimer’s disease

AD is a chronic neurodegenerative disease characterized by progressive cognitive and memory deficits, as characterized by massive death and loss of central cholinergic neurons, hyperphosphorylation of Tau proteins to form neurofibrillary tangles and deposition of β-amyloid (Aβ), which leads to damage to cholinergic nerve cells, decreased levels of choline acetyltransferase (ChAT) and decreased acetylcholine transmitter synthesis is reduced (Busche and Hyman, 2020). PNS has been shown to be advantageous in ameliorating neurological disorders such as Alzheimer’s, improving learning and memory deficits (Liu S. et al., 2024). Its treatment mainly targets Aβ production deposition, Tau protein phosphorylation and cholinergic nervous system.

PNS can regulate the expression of amyloid beta precursor protein (APP) gene in the brain at the transcriptional level, activate PKA/CREB signaling, promote APP to shear with α-secretase and inhibit γ-secretase activity (Fang et al., 2012), upregulate the expression of ADAM9 mRNA, downregulate the expression of β-secretase (BACE1) protein in the brain, and reduce Aβ generation, and improve the learning and memory ability of rapid aging model mice (Huang et al., 2014). R1 intervention in APP/PS1 double transgenic AD model mice, by inhibiting K^+^ channel activity may contribute to the reduction of spiking thresholds, promote the increase of LTP and ultimately regulate neuronal excitability, which can improve their learning and memory ability, and have a certain restorative effect on degenerative neuropathy (Yan et al., 2014). Rb1 inhibits ROS production, increases Bcl-2/Bax and inhibits caspase-3 activity, and maintains the balance of oxidative stress in Aβ-induced neuronal cells to exert a neuroprotective effect (Xie et al., 2010; Shekhar et al., 2018). R1 also increases Aβ_25-35_cell viability in cultured PC12 neurons, reduces oxidative damage, restores mitochondrial membrane potential and activates the MAPK signaling pathway to counteract the effects of Aβ (Ma et al., 2014). Studies have shown that elevated levels of Nav1.1α are associated with cognitive deficits in mice (Corbett et al., 2013), R1 induced inhibition of BACE1 activity by altering the number and/or distribution of voltage-gated sodium channel (Nav) members, modulating the enzymatic cleavage of Navβ2 to regulate sodium currents, correcting the aberrant distribution of Nav1.1α, ameliorating abnormal neuronal overexcitation and memory deficits, promoting neuronal repair and cognitive improvement in AD mice (Hu et al., 2020).

The pathogenesis of AD is associated with the dephosphorylation of phosphatases such as glycogen synthase kinase-3 and protein phosphatase-2A (PP2A). Rb1 was found to protect against aluminum-induced neurotoxicity by preventing Tau protein phosphorylation through the regulation of p-GSK3 and PP2A levels (Zhao et al., 2013). PNS can also protect and improve the function of the central cholinergic system by improving the quantity and quality of cellular survival, and by increasing the content and activity of the enzyme ChAT through the improvement and repair of damaged neurons. The central cholinergic system.

*In vitro* and *in vivo* experiments have demonstrated that PNS can alleviate diseases caused by AD risk factors by modulating HPA axis disorders, lowering excess free FFA levels, improving insulin resistance, and promoting vascular wall thickening to reduce Aβ accumulation and Tau hyperphosphorylation in the brain (Wu et al., 2024). PNS enhances the PINK1/Parkin pathway, promotes mitophagy in the hippocampus, reduces brain oxidative stress in SAMP8 mice, and increases PC12 cell viability, elevating LC3II/I protein levels while decreasing p62 protein and OPTN, thereby alleviating neuronal damage in AD (Jiang et al., 2022; Yang et al., 2024b). PNS can alleviate the disease caused by AD risk factors through anti-inflammatory and anti-cellular effects, as well as through Tau excess phosphorylation. PNS can reduce glutamate metabolism dysfunction induced by the chemokine CC motif ligand 2, inhibit oxidative stress induced by the over-activation of the N-methyl-D-aspartate receptor, lower the Bax/BCL-2 ratio and inhibit apoptosis by reducing the expression of caspases-3, 8, and 9, and thus alleviate cognitive deficits in rats (Zhou et al., 2020).

R1 can alleviate impaired learning and memory in SD mice by modulating the melatonin receptor type 1A-mediated PI3K/Akt/mTOR signaling pathway to reduce excessive autophagy and apoptosis in hippocampal neurons (Huang G. et al., 2017; Cao et al., 2021). R2 is able to inhibit apoptosis through the miR-27a/SOX8/β-catenin axis expression, attenuate Aβ_25-35_-triggered neuronal apoptosis and inflammation, and inhibit cortical neuronal apoptosis and attenuate inflammation in AD rats as a way to enhance cognitive function and improve AD symptoms in AD rats (Hu et al., 2021); It can also prevent isoflurane-induced learning and cognitive impairments by promoting miR-29a expression and preventing inflammatory responses (Wang et al., 2022).

In conclusion, Aβ is a key initiator of the AD pathological cascade. PNS promotes mitochondrial autophagy, restores mitochondrial membrane potential, and reduces oxidative stress damage in the hippocampus through PINK1/Parkin and MAPK signaling pathways; regulates excessive autophagy and apoptosis in hippocampal neurons via PI3K/Akt/mTOR signaling pathways; and downregulates BACE1 protein expression in the brain through PKA/CREB signaling. BACE1 protein expression as a way to reduce Aβ production. PNS also prevents Tau protein phosphorylation by regulating p-GSK3 and PP2A. PNS plays a critical role in AD-induced neurological dysfunction by inhibiting neuroinflammation, oxidative stress, and regulating mitochondrial autophagy and apoptosis, thereby reducing Aβ production and preventing Tau protein phosphorylation.

### 6.2 PNS and Parkinson’s disease (PD)

PD is a neurodegenerative disorder in which the balance between dopamine and acetylcholine is dysregulated due to degenerative changes in dopamine (DA) neurons in the dense nigrostriatal region of the midbrain (Marino et al., 2020). Associated with multiple factors such as aging, genetic susceptibility and environmental exposure (Gao and Hong, 2011).

PNS may attenuate microglia-mediated neuroinflammation through the P2Y2R/PI3K/AKT/NF-κB signaling pathway, inhibit the production of inflammatory markers, such as IL-1β, IL-6, and TNF-α, in LPS-stimulated BV-2 cells, and attenuate behavioral deficits and excessive microglial activation in PD model mice, thereby reducing the progression of PD (Wu et al., 2025). Rg3 has the ability to ameliorate dopamine neuron neurodegeneration in a 6-hydroxydopamine (6-OHDA)-induced PD model through inhibition of apoptotic mediators and stimulation of antioxidant enzymes (Chalorak et al., 2021). Mitochondrial dysfunction remains a key mechanism in a variety of neurodegenerative diseases, in which autophagy receptors are recruited by the ubiquitin kinase PINK1 to induce mitochondrial autophagy. PINK1 and PARKIN-related autophagy processes are able to modulate neurodegeneration and neuroinflammation by removing dysfunctional mitochondria, controlling mtDNA release, or promoting neuroprotective and anti-inflammatory manifestations (Lazarou et al., 2015; Quinn et al., 2020; Olagunju et al., 2023).

Rg1 can exert a neuroprotective effect on 6-OHDA induced dopaminergic neurons in the substantia nigra striata damaged dopaminergic neurons in a rat model of PD through the insulin-like growth factor-I receptor (IGF-I) signaling pathway (Xu et al., 2009). Ginsenoside triol saponin (PTS) from Panax notoginseng inhibited 1-methyl-4-phenyl-1,2,3,6-tetrahydropyridine (MPTP)-induced neurotoxicity in PD mice, and further suppressed MPTP-induced neuronal death in the substantia nigra compacta by increasing Trx-1 expression, inhibiting cyclooxygenase-2 overexpression, and inhibiting mitochondrial-mediated apoptosis, and then could effectively PD (Luo et al., 2011).

Studies have shown that dopamine neurons lack degeneration, neuroinflammation, mitochondrial dysfunction and oxidative stress are the characteristics of its pathogenesis, and PNS can alleviate dopamine neuron damage and apoptosis through P2Y2R/PI3K/AKT/NF-κB and IGF-I signaling pathways by exerting anti-inflammatory and antioxidant effects, as well as through the autophagy process mediated by PINK1 and PARKIN to effectively ameliorate PD. effectively improve PD.

### 6.3 PNS and depression

Depression is a psychoaffective disorder characterized by persistent low mood, loss of interest, and impairment of cognitive function (Dean and Keshavan, 2017). Current evidence suggests that the onset of depression may be associated with reduced neurotransmitter secretion (Jeon and Kim, 2016). In PNS, saponins R1, Rg1, Rb1, Rc, and Rb3 are important representative components of antidepressants (Cui et al., 2012), which may be involved in the regulation of neurotransmitter mechanisms (5-HT, DA, and NE), modulation of gamma-aminobutyric acid (GABA) neurotransmission, the glutamatergic system, the HPA axis, BDNF, and their intracellular signaling in the CNS pathways produce neuronal protection and regulate nerve cell activity and secretion, thus exerting antidepressant and anxiolytic effects (Xie et al., 2018a).

PNS modulates calmodulin kinase channels (Ca^2+^/CaM/CaMK), reduces internal concentrations of Ca^2+^in neuronal cells, and promotes the release of the neurotransmitters 5-HT, NE, and DA to reduce depressive behavior (Albert and Fiori, 2014; Zhang et al., 2016). It has been shown that neuroinflammation is a key pathological mechanism contributing to depressive-like behaviors (O'Connor et al., 2009), High levels of pro-inflammatory cytokines (IL- 6, IL-1β, TNF- α ) and C-reactive protein in peripheral serum of patients with major depressive disorder (Young et al., 2014), and by injection of LPS into lateral ventricles induced by a mouse neurological Inflammation model of mice induced by injection of LPS into the lateral ventricle produces obvious depressive-like behavior, and the indicators of neuroinflammation in the brain (Iba-1, TNF- α , IL-1β, and IL-6) are significantly increased, and the depression-like behavior of the mice is significantly improved after treatment with R1, suggesting that R1 can play an antidepressant role by improving the neuroinflammation of the brain. R1 can also improve the chronic stress-induced chronic inflammation in the rat through activation of the PI3K/AKT/NF-κB pathway. Chronic stress-induced chronic unpredictable mild stress, decreasing the levels of Glu and Asp and increasing the levels of GABA and Tau in the hippocampus, and inhibiting depressive behaviors in rats (Wu et al., 2012; Zhan et al., 2022). Rg1 can exert antidepressant effects by activating the neurotrophic factor signaling pathway and promoting hippocampal neurogenesis through the BDNF- TrkB signaling pathway (Jiang et al., 2012).

In an antidepressant-like activity assay between ginsenoside Rb3 and its four deglycosylated derivatives, Rg3, Rh2, Compound K (C-K), and PPD, only C-K and Rg_3_, which are the active deglycosylated derivatives of Rb_3_, exerted antidepressant-like effects in mice in the forced-swimming test (FST) and the tail-suspension test (TST), and in particular Rg3 was more effective in restoring brain monocorticolysis than Rb3. Rb3 is more effective in restoring brain monoamine neurotransmitter 5-HT, decreasing dopamine levels, increasing norepinephrine levels, etc. exerting antidepressant-like effects (Zhang et al., 2016). Rg3 significantly reduces LPS-induced plasma levels of IL-6 and TNF-α, restores systemic homeostasis of tryptophan-kynurenine metabolism, and effectively ameliorates immune-activation-induced depressive-like behavior (Kang et al., 2017).

PNS can improve depressive-like behavior by multifaceted modulation of monoamine neurotransmitters, neuroinflammation, activation of the BDNF-TrkB signaling pathway, inhibition of oxidative stress, and regulation of the HPA axis.

### 6.4 PNS and traumatic brain injury (TBI)

TBI is a severe traumatic brain injury consisting of both primary and secondary damage; the primary damage is irreversible, while the secondary damage consists of a series of physiological and molecular biological changes such as oxidative stress, inflammatory response, mitochondrial apoptosis, oxygen free radical production, and disruption of the BBB (Jiang et al., 2019).

In a rat TBI model, R1 administration (intraperitoneal injection of 40 mg/kg) reduced the expression levels of ERK and p-RSK1, improved neuronal apoptosis in brain injury by inhibiting the ERK signaling pathway, reduced neurological deficits after TBI, and inhibited the expression of pro-inflammatory factors (Pei et al., 2023). Cerebral edema, as one of the serious secondary pathological changes after TBI, can lead to increased intracranial pressure and corresponding decreased cerebral perfusion, which is further aggravated leading to brain tissue injury (Lutton et al., 2017). PNS can improve microcirculation around the ischemic area of hematoma, promote the absorption of hematoma, and slow down and inhibit the development of cerebral edema. When using PNS to treat cerebral edema, the use of PNS in the early stage of cerebral hemorrhage or TBI may aggravate cerebral edema and increase neurological deficit scores, so PNS should be used with caution in treating patients with large numbers of cerebral hemorrhages in the early stage of cerebral hemorrhage or TBI (Nie et al., 2006).

## 7 Single-agent of PNS clinical application in neurological disorders

Currently, PNS is approved for clinical use in China by the China Drug Administration in various dosage forms. PNS injections are mainly used to treat neurological diseases such as cerebral hemorrhage and cerebral ischemia (see Table 4).

**TABLE 4:** Single-agent of panax notoginseng saponins clinical application in neurological disorders.

Disease	Study type	Panax notoginseng saponins form, dose and therapy	Control group	Efficacy and results	Adverse events	References
HS	RCT (n = 63)	Xueshuantong Injection	Regular treatment plus 250 mL saline	Hematoma and inflammatory reactions, NIHSS score, leukocyte count, CRP↓	No obvious adverse reactions	Gao et al. (2012)
HS	Meta-analysis (20 RCTs)	(Xuesaitong PNS freeze-dry powder, Xueshuantong, Xuesaitong, Lulutong) injections	Western medicine treatment	Barthel index Barthel↑, neurological deficit score, mortality rate, intracerebral hematoma volume, intracerebral edema volume↓	skin rash	Xu et al. (2014)
IS	RCT (n = 3072)	Xuesaitong soft capsules, (120 mg orally twice daily)	placebo (120 mg orally twice daily)	Functional independence↑	intracranial hemorrhage	Wu et al. (2023)
IS	Meta-analysis	PNS injections (including Xuesaitong injection and Xueshuantong injection)	Western medicine treatment	neurological deficits and activities of daily living↑	allergy, gastrointestinal reaction, dizziness	Liu Y et al. (2024b)
IS IS Cerebral Infarction	Meta-analysis (17 RCTs) Meta-analysis (12 RCTs) Meta-analysis (23 RCTs)	Xuesaitong soft capsule Xuesaitong injection combined with western medicines Xuesaitong injection	Western medicine treatment Western medicine treatmen Western medicine treatment	crooked mouth and tongue, and dizziness↓ nerve function and the quality of life↑ plasma viscosity, fibrinogen level, whole blood high shear viscosity, and whole blood low shear viscosity↓ degree of neurological deficit, plasma viscosity, blood lipids and lipid peroxidation products ↓	mild gastrointestinal damage pruritus, gastrointestinal discomfort, dizziness and headache No obvious adverse reactions	Gao Y et al. (2022b) Feng et al. (2021b) Zhang et al. (2015)

IS, ischemic stroke; HS, Hemorrhagic stroke; CRP, C-reactive protein; IGF-1, insulin like growth factor-1; IL-6, interleukin-6; GCS, Glasgow Coma Scale; MMSE, Mini-Mental State Examination; NIHSS, National Institute of Health stroke scale.

Clinical research evidence indicates that single-agent preparations of PNS (mainly injections or soft capsules of Xuesaitong and Xueshuantong) show positive effects in the treatment of hemorrhagic stroke, ischemic stroke, and related cerebral infarction. Multiple randomized controlled trials and meta-analyses have shown that their application can effectively improve patients’ neurological deficit scores, activities of daily living (Barthel index), promote hematoma absorption, reduce cerebral edema, and improve hemorheological indices. Overall, single-agent treatment with PNS has significant clinical efficacy and good safety in cerebrovascular diseases (especially ischemic and hemorrhagic strokes), providing strong evidence for its widespread application.

## 8 Treatment of neurotoxicity

Neurotoxicity refers to damage to the structure or function of the nervous system caused by exogenous chemicals or biological factors, including excessive production of free radicals and decreased activity of antioxidant enzymes; inflammatory factors inducing neuronal damage and death; apoptosis; and imbalance of calcium ions and neurotransmitters. This in turn affects neuronal, glial cell, synaptic transmission or overall neural circuit function.

In the study of H_2_O_2_ induced neurotoxicity and led to PC12 cell death, Rb1 was able to effectively increase cell viability and inhibit apoptosis, thus protecting PC12 cells from toxic effects (Zeng et al., 2015). In addition, Rb1 can prevent Tau protein phosphorylation and thus aluminum-induced neurotoxicity by regulating the levels of p-GSK3 and PP2A, further exerting neuroprotective effects (Zhao et al., 2013). It can also block spatial and cognitive deficits induced by acrylamide administration, attenuate acrylamide-induced neurotoxicity by up-regulating PC12 cells and regulating autophagy by Trx-1 (Wang et al., 2020). R1 can rescue apoptosis and attenuate bupivacaine-induced neurotoxicity by activating Jak1/Stat3 pathway and triggering upregulation of Mcl1. Induced neurotoxicity and reduce neuronal damage (Yang et al., 2024c). R1 can block the downregulation of Bcl-2 and the upregulation of Bax, which can inhibit the increase of intracellular free Ca^2+^, reduce the overproduction of intracellular ROS, and prevent the depolarization of the mitochondrial membrane potential, thereby protecting the cortical neuronal cells against neurotoxicity caused by glutamate (Glu) exposure in mice (Gu et al., 2009). PNS attenuates sevoflurane-induced neuronal cytotoxicity, protects neuronal cells, and promotes neuronal regeneration by modulating the AKT signaling pathway (Yang et al., 2017). PTS from Panax notoginseng inhibits MPTP induced neurotoxicity in PD mice, attenuates neurotoxicity and nourishes nerves through multi-pharmacological properties, such as antioxidant, inhibition of inflammatory response, and mitochondria-mediated apoptosis (Luo et al., 2011).

In conclusion, in the face of different types of neurotoxicity, PNS can regulate neuronal cells through multiple mechanisms and multiple cell signaling pathways. Therefore, future studies need more experiments to evaluate the potential efficacy and safety of PNS in clinical use for the treatment of neurological disorders.

## 9 Adverse events

Side effects of PNS are relatively rare in clinical practice. In a systematic evaluation, adverse reactions associated with Xuesaitong in combination with conventional medications included only 0.27% rash (1/363), and no serious adverse events were reported (Yang et al., 2013). In a randomized clinical trial of efficacy and safety, the incidence of serious adverse events in the PNS (Xuesaitong soft capsules) group at 3 months was 1.0% (15 of 1488 patients), and other secondary safety outcomes, including symptomatic intracranial hemorrhage (0.1%), all-cause mortality (0.1%), and adverse events (2.8%) were not reported (Wu et al., 2023). In a systematic evaluation and meta-analysis of the results of intravenous thrombolysis after the use of PNS injection for acute ischemic stroke, seven studies reported the occurrence of adverse events and five reported the occurrence of hemorrhagic transformation, with two studies reporting no adverse events and three reporting specific adverse events (*n* = 527), which were mild and self-limiting and consisted mainly of allergies, gastrointestinal reactions, and Dizziness (Liu Y. et al., 2024). In another study of the efficacy and safety of PNS in patients with acute ischemic stroke, the incidence of adverse events was very low, with nausea, dizziness, and skin irritation being the most commonly reported adverse events. In a study of the effect of age on the efficacy and safety of PNS for acute ischemic stroke, no symptomatic intracranial hemorrhage or all-cause mortality was observed, regardless of age. In summary, PNS has shown good safety in combination with conventional treatment (see Table 5).

**TABLE 5:** The incidence of adverse reactions with PNS for cerebrovascular neurological disorders.

Adverse events	The incidence of adverse reactions (experimental)	The incidence of adverse reactions (control)	References
Rash	1/363	Compound Danshen injection	Yang et al. (2013)
		Western medicine treatment	
Intracranial hemorrhage	15/1488	16/1482	Wu et al. (2023)
hemorrhagic transformation	3/263	4/263	Liu Y et al. (2024b)

Regarding the strength and consistency of clinical evidence for PNS in different cerebrovascular and neurological diseases, the clinical application evidence was integrated, analyzed, and systematically evaluated, thereby revealing the current credibility of PNS in its research field and the direction for future studies (see Table 6).

**TABLE 6:** Analysis of evidence integration and transformation potential.

Disease	Strength of clinical evidence	Consistency of evidence	Key basis and limitations
IS	Medium-High	Consistent	Multiple mechanisms have been confirmed, and several RCTs and meta-analyses support its improvement of neurological deficits and daily living abilities, but standardized dosing and long-term benefits need to be clarified
HS	Medium	Mostly consistent	Animal models show promotion of hematoma absorption, and clinical studies are effective, but some studies suggest that early use may worsen cerebral edema, so the risk-benefit needs to be precisely defined
AD	Low	Insufficient data	Preclinical mechanistic studies are extensive and diverse, but there is currently a lack of high-quality clinical research, resulting in high translational uncertainty

Precise drug development should target specific potential components (such as the procoagulant factor Ft1); further clarify their pharmacokinetic-pharmacodynamic relationships in secondary prevention of acute ischemic stroke; evaluate their safety in real-world settings in combination with standard antithrombotic drugs; and develop innovative formulation strategies aimed at enhancing blood-brain barrier delivery efficiency.

## 10 Conclusion

PNS, as the main active ingredient of Panax notoginseng, has multi-target and multi-pathway neuroprotective effects in the treatment of cerebrovascular and neurological diseases, and its biological activity mainly exerts cerebrovascular and neurological protective effects through anti-inflammatory, antioxidant, anti-apoptosis, immunomodulation, etc. to improve microcirculation, promote nerve regeneration, and inhibit protein aggregation, etc. Many clinical studies have provided corresponding results for the use of PNS in the treatment of cerebrovascular-like and neurodegenerative diseases, etc. Many clinical studies have provided corresponding evidence for the treatment of PNS in cerebrovascular and neurodegenerative diseases, confirming that PNS is a potential neuroprotective agent and can be used as an adjunctive treatment for cerebrovascular and neurodegenerative diseases. However, the low oral bioavailability and limited penetration of BBB during drug delivery require further optimization of drug delivery strategies and strengthening of clinical translational research.
