# Correction: The role of panax notoginseng saponins in cerebrovascular neurological disorders: an overview of mechanisms and functions

**DOI:** 10.3389/fphar.2025.1750932

**Published:** 2025-12-03

**Authors:** Bin Wang, Xu Gao, Yali Zhang, Yuhua Xiao, Tong Chen, Zhouying Shi, Yue Yuan, Ping Li

**Affiliations:** 1 College of Traditional Chinese Medicine, Changchun University of Chinese Medicine, Changchun, China; 2 College of Basic Medicine, Changchun University of Chinese Medicine, Changchun, China; 3 College of Nursing, Changchun University of Chinese Medicine, Changchun, China

**Keywords:** panax notoginseng saponins, cerebrovascular, neurological diseases, pharmacologic effects, mechanism of action

An incorrect **Funding statement** was provided.

The funder “the National Traditional Chinese Medicine Clinical Excellence Talent Advanced Study Program (National Administration of TCM Personnel and Education Letter [2022] No. 239)” was erroneously omitted.

The original article has been updated.

